# A Case Report of Dental Implants and Site Augmentation in a Patient with Erosive Lichen Planus

**DOI:** 10.1155/2022/9508580

**Published:** 2022-05-16

**Authors:** Abdulrahman Aseri

**Affiliations:** Department of Preventive Dental Sciences, Faculty of Dentistry, Najran University, Saudi Arabia

## Abstract

Lichen planus (LP) is an autoimmune inflammatory disease that affects oral mucosal tissue, leading to complications in patients treated with dental implant therapy. This case report discusses the clinical management of a patient diagnosed with an erosive type of LP. After disease management, the patient was treated with dental implants in the upper and lower jaws with augmentation procedures around the implants. All implants were loaded after a three-month period of healing. Slight bone loss was observed in the upper premolar area following an exaggerated soft tissue response to the augmentation procedure. Within one year of implant placement, no additional complications were encountered.

## 1. Introduction

Oral lichen planus is an inflammatory autoimmune mucocutaneous disease that affects the skin, oral mucosa, genitalia, scalp, and nails. It mainly affects people in their middle age with an estimated prevalence ranging from 0.1 to 2.2% of the population, with 3 : 2 female predilection. LP can be present orally in several forms; the most common forms are reticular and erosive LP [1–3].

Dental implant prostheses have been widely used to effectively replace missing teeth with a survival rate ranging from 93 to 95% [4]. Multiple factors can dictate dental implant success or failure, including systemic health, history of periodontal disease, quality, and quantity of hard and soft tissues [5]. Following a dental extraction, the alveolar ridge can undergo up to 50% dimensional changes within 12 months [6]. Even with ridge preservation techniques, the alveolar ridge cannot be preserved entirely [7, 8]. Additional hard and soft tissue augmentations around the implant can aid in maintaining stable alveolar bone and provide healthier peri-implant tissues [9].

LP in its erosive form can affect the quality of soft tissue. Few studies have looked at the effect of oral mucosal lesions, namely LP, on the success of dental implants [10, 11]. Furthermore, there is a scarcity of evidence investigating the effect of LP on soft tissue quality and tissue response to surgical implant therapy. These surgeries include guided bone regeneration around the alveolar ridge or simultaneously with implant placement. These surgical procedures require flap manipulation at the mucosal level to achieve tension-free primary closure [12]. This case report was aimed at assessing and discussing the surgical management of a patient with LP treated with dental implant therapy with implant site augmentation.

## 2. Materials and Methods

**Figure 1:**
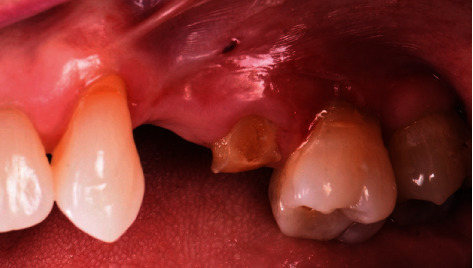
Desquamative erythematous lesion in the vestibular area upper left quadrant.

**Figure 2:**
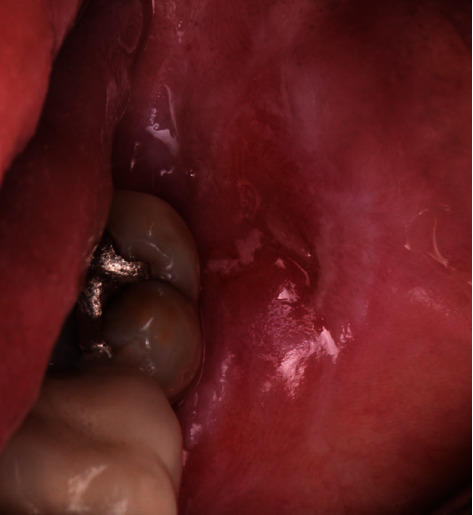
Lichenoid lesion related to tooth #37.

**Figure 3:**
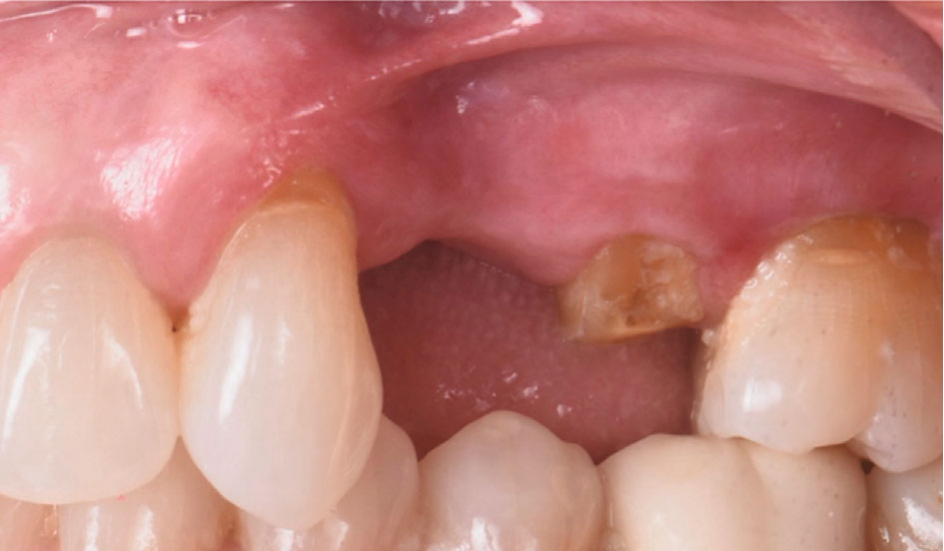
Improved soft tissue response to topical corticosteroid therapy two months after diagnosis.

**Figure 4:**
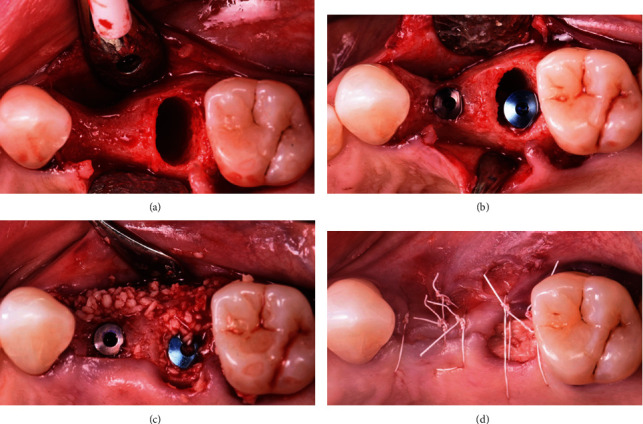
The sequence of the surgical management of the upper left quadrant. (a) After tooth extraction and ridge deficiency is noticed at the 1^st^ premolar site. (b) Implant placement at #25 and 24. (c) Allograft bone material is used to fill the buccal gap at the extraction socket and augment the buccal ridge at #24. (d) Upon flap closure, a tissue tear was noticed at the mucosal area of the site.

**Figure 5:**
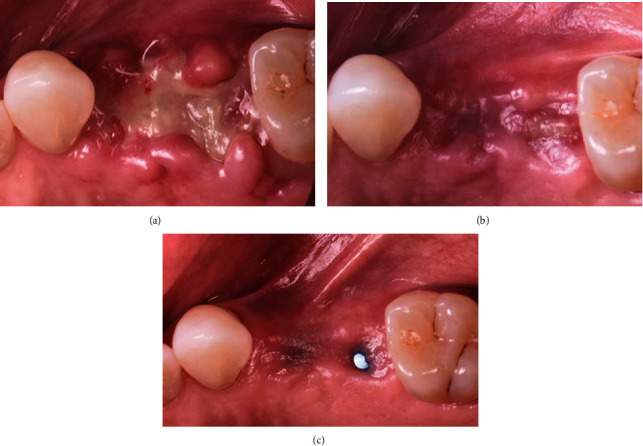
(a) Eight days of follow-up presented with exaggerated tissue response to the surgery. (b) Four weeks postoperative with better soft tissue response. (c) Eight weeks postoperative with thin, soft tissue covering the implants.

**Figure 6:**
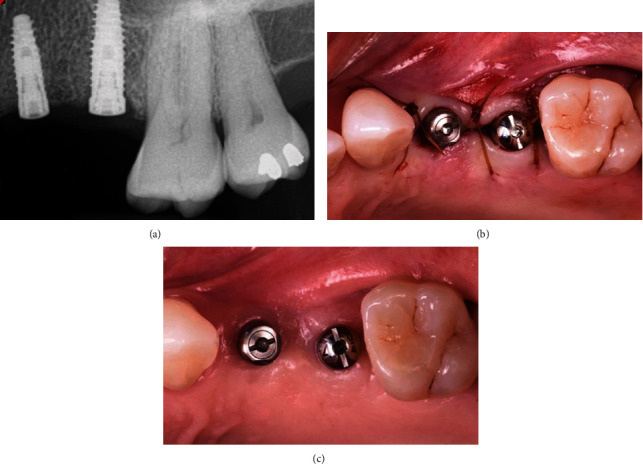
(a) At three months postimplant placement, the radiograph showed slight crestal bone loss. (b) Implants exposure with healing abutments. (c) Two weeks healing with better soft tissue healing and healthier-looking tissue.

A 65-year-old female patient was referred to the periodontics clinic to evaluate extraction of the hopeless upper left second premolar and dental implants placement. Medical history showed high cholesterol and hypothyroidism under control of medication. Upon clinical examination, the patient had fair oral hygiene with a history of regular visits to the dental office for maintenance every six months. Erythematous and ulcerated mucosal tissue were observed on the buccal vestibule of the upper left and lower left quadrants (Figures 1 and 2). The patient reported sensitivity to citrus fruits and spices in these specific oral areas. A partially edentulous area was seen on radiographs at the upper left first premolar and lower right, as well as a retained root tip at the upper left second premolar. The patient was referred to an oral medicine specialist to manage the oral lesion. Two punch biopsies were taken from the upper left quadrant; the first biopsy was placed in formalin solution for H&E staining, and the second was submitted in Michel's solution for an immunofluorescence test. The diagnosis of erosive lichen planus was confirmed by a pathology report. After that, the patient began applying fluocinonide 0.05 percent gel three times a day. Meanwhile, dental procedures like scaling, polishing, and oral hygiene instructions were conducted carefully while monitoring the status of the soft tissue response to the corticosteroid therapy. Two months after systemic and local treatments, the patient presented with an improved soft tissue status and healthier-looking tissue (Figure 3). Clearance for dental implant therapy was obtained from the oral medicine specialist. A cone beam CT scan revealed adequate bone height and width to place an immediate implant at tooth #25 and implant placement at #24 with contoured bone augmentation. Before surgery, verbal and written consent was obtained, and the patient was premeditated with 1 g of amoxicillin one hour before the surgery to minimize the risk of complications or implant failure [13]. Tooth #25 was then extracted as minimally traumatic as possible, followed by curettage to ensure that all granulomatous tissues were removed. The socket was then thoroughly irrigated with normal saline. The immediate implant (Biomet 3i), #25, was placed in the freshly extracted socket, and #24 was placed in the healed ridge with buccal ridge deficiency. The buccal gap at the extraction socket and the buccal concavity at the first premolar site were grafted with freeze-dried bone allograft (Oragraft, Lifenet Health) and covered with collagen membrane (Ossix plus, ColBar Life Sciences Ltd). A periosteal releasing incision was made deep into the buccal flap to achieve tension-free primary closure. The flap was sutured using 4-0 expanded polytetrafluoroethylene. The tissue was difficult to suture at this point, and tears at the buccal mucosa were noticed upon tightening the sutures (Figure 4). The patient was then given verbal and written postoperative instructions, and medication was prescribed 500 mg of amoxicillin and nonsteroidal anti-inflammatory drugs as an analgesic, ibuprofen 400 mg. The patient presented with an exaggerated response to the procedure eight days after implant placement, as sloughed tissue covered the surgical site. At this stage, it was decided to remove the sutures after irrigating with normal saline. Healing was within normal limits after four weeks, with no sign of inflammation except at the area of the second premolar, where slightly ulcerated tissues were still clinically evident. On eight weeks follow-up, healing appeared to be better with around 2 × 2 mm soft tissue exposure of cover screw at the site of the second premolar (Figure 5). The patient was then scheduled for a second-stage appointment. During that procedure, minimal flap manipulation was used to minimize further exaggerated tissue response. After the second-stage surgery, healing was uneventful. The tissue appeared firm, pink in color, and free of inflammation (Figure 6). The same procedure was performed for tooth #46, where the implant was placed in a healed ridge with adequate bone and soft tissue thickness. When compared to the other site, no abnormal tissue response was observed. Furthermore, no flare-ups were detected during the subsequent appointments, and the patient was referred to the restorative dentist for a final impression and crown fabrication.

## 3. Discussion

In this case of erosive LP, the patient was treated with dentoalveolar implant placement in the upper left and lower right quadrants with variable degrees of tissue response to the surgical therapy. Even though few studies have examined the success of implant placement in patients with LP, when the disease is appropriately managed by a specialist, it can be assumed that implant placement and site augmentation can be a viable approach to restoring missing dentition in such patients [11].

A total of fourteen patients who received 1-15 implants were investigated over a two-year period in a retrospective study to investigate whether oral LP affected the success of dental implant therapy. The study suggested that a well-treated LP does not appear to negatively impact the success of dental implants [14]. In a cross-sectional study evaluating clinical parameters of 16 patients diagnosed with LP who received dental implants loaded for at least a year, the clinical parameters were peri-implant mucositis, peri-implantitis, bone loss, pain, and bleeding. The study did not find any statistically significant difference in all these parameters when comparing dental implants placed in patients with LP and those without LP [15].

Several studies investigated the effect of soft tissue thickness on the stability and health of peri-implant bone [16, 17]. Linkevicius et al. in 2015 reported in a study evaluating implant placement with platform switching prosthetic design in two groups of thick and thin soft tissues. The study found that implants placed in thin soft tissue had more bone loss when compared to those placed in thick soft tissue after one year of loading [18]. One of the clinical challenges encountered with patients with LP is the risk of exaggerated oral tissue response to surgical therapy, which may have an impact on the quality and quantity of soft tissue. This was a clear finding in the patient in this case report where crestal bone loss was evident three months after placing the implant in the upper premolar site. This observation could be attributed to the soft tissue exposure that eventually affected the soft tissue thickness around the implant.

In a systematic review, Chrcanovic et al. reported a low failure rate of implants placed in patients with LP (2.7%). The study recommended that dental implant surgery be performed in the disease remission stage in a site with no desquamative gingivitis to avoid peri-implant soft tissue inflammation [11]. In the case presented in this report, surgery was postponed until better soft tissue quality was evident after disease management and adequate plaque control were achieved by the patient.

The most common types of LP are the reticular and erythematous erosive [1]. Many therapeutic approaches have been documented for the treatment of LP, like glucocorticoids, calcineurin derivatives, vitamins A and E, and laser therapy [19] [20]. Following a referral to an oral medicine specialist for disease management, a topical corticosteroid was recommended to ensure that the patient was asymptomatic and in no discomfort prior to any surgical intervention.

Good plaque control should be an essential part of disease management in patients diagnosed with LP to eliminate soft tissue inflammation triggering factors. These patients may experience sensitivity to regular plaque control regimens and some toothpaste, which may lead to suboptimal oral hygiene and negatively impact the patient's life [21] [22]. As a result, a structured plaque control regimen and products can reduce the severity of LP lesions while also enhancing the individuals' oral health-related quality of life [23].

López-Jornet et al. evaluated the quality of life of 3 groups of patients: implants with LP, LP patients with no implant, and implants with no LP. This study found a difference between the three groups in which the patients with LP and no implant experienced the worst quality of life compared to the other two groups. The author attributed that the presence of dental implant prosthesis may have a positive impact on mastication and esthetic whether the patient is diagnosed with LP or not [15].

Few studies in the literature evaluated the effect of LP on dental implant therapy. Future researches need to investigate the long-term impact of LP on the survival and success of dental implants, utilizing larger samples and a longer follow-up period.

## 4. Conclusion

This case report details the treatment of a female patient with erosive lichen planus who underwent dental implants and site augmentation surgery. Dental implant therapy can be a viable treatment option for replacing missing teeth in patients with LP, given that the disease is in remission and the surgical procedure is performed on a good quality soft tissue.
